# Resveratrol inhibits Cdk5 activity through regulation of p35 expression

**DOI:** 10.1186/1744-8069-7-49

**Published:** 2011-07-07

**Authors:** Elias Utreras, Anita Terse, Jason Keller, Michael J Iadarola, Ashok B Kulkarni

**Affiliations:** 1 Functional Genomic Section, Laboratory of Cell and Developmental Biology, National Institute of Dental and Craniofacial Research, National Institutes of Health, 30 Convent Dr., Bldg. 30, Rm. 130, MSC 4395, Bethesda, MD 20892, USA; 2 Neurobiology and Pain Therapeutics Section, National Institute of Dental and Craniofacial Research, National Institutes of Health, 49 Convent Drive, MSC 4410 Bethesda, MD 20892, USA

**Keywords:** Cdk5, resveratrol, TNF-α, pain, Egr-1, ERK1/2, analgesic

## Abstract

**Background:**

We have previously reported that cyclin-dependent kinase 5 (Cdk5) participates in the regulation of nociceptive signaling. Through activation of the ERK1/2 pathway, Tumor Necrosis Factor-α (TNF-α) induces expression of Egr-1. This results in the sustained and robust expression of p35, a coactivator of Cdk5, in PC12 cells, thereby increasing Cdk5 kinase activity. The aim of our present study was to test whether resveratrol, a polyphenolic compound with known analgesic activity, can regulate Cdk5/p35 activity.

**Results:**

Here we used a cell-based assay in which a p35 promoter-luciferase construct was stably transfected in PC12 cells. Our studies demonstrate that resveratrol inhibits p35 promoter activity and also blocks the TNF-α mediated increase in Cdk5 activity in PC12 cells. Resveratrol also inhibits p35 expression and blocks the TNF-α mediated increase in Cdk5 activity in DRG neurons. In the presence of resveratrol, the MEK inhibitor decreased p35 promoter activity, whereas the inhibitors of p38 MAPK, JNK and NF-κB increased p35 promoter activity, indicating that these pathways regulate p35 expression differently. The TNF-α-mediated increase in Egr-1 expression was decreased by resveratrol treatment with a concomitant reduction in p35 expression and protein levels, resulting in reduced Cdk5 kinase activity.

**Conclusions:**

We demonstrate here that resveratrol regulates p35 promoter activity in PC12 cells and DRG neurons. Most importantly, resveratrol blocks the TNF-α-mediated increase in p35 promoter activity, thereby reducing p35 expression and subsequent Cdk5 kinase activity. This new molecular mechanism adds to the known analgesic effects of resveratrol and confirms the need for identifying new analgesics based on their ability to inhibit Cdk5 activity for effective treatment of pain.

## Background

Resveratrol, a naturally occurring polyphenol found mainly in red wine, grapes and berries, has been shown to have many therapeutic values. It is known to protect against heart disease and cancers, promote anti-aging effects, suppress neuronal degeneration and also act as an analgesic [1-5]. Earlier studies indicated that intra-peritoneal administration of resveratrol decreases hyperalgesia in the rat model of inflammatory pain, which was induced by carrageenan injection in the hind paw [6], and this was attributed to the previously reported inhibitory effects of resveratrol on cyclooxygenase (COX)-2 expression [7]. Intra-cerebral injections of resveratrol also suppressed hyperalgesia in the rat model of thermal pain with a concomitant inhibition of COX-1 and COX-2 [8]. Similar analgesic effects of resveratrol were also observed in the rat model of diabetic neuropathic pain [9,10]. All of these studies indicate that resveratrol has analgesic properties against acute and chronic pain that is triggered either by inflammation, heat or a diabetic condition. Although some of these studies have linked this analgesic action of resveratrol with altered expression of TNF-α and nitric oxide in the diabetic rat model [10] and reduced expression of COX-2 in the inflammatory pain model [11], there is a lack of clear understanding as to how resveratrol brings about its analgesic action.

We have recently reported a novel role of Cdk5 in pain signaling [12,13]. Cdk5 is a proline-directed serine/threonine protein kinase that belongs to the family of cyclin-dependent kinases. It is expressed in all tissues, but it is functionally active mainly in the neurons where its activators, p35 and p39, are predominantly expressed. We and others have previously reported that expression of Cdk5 and p35, as well as Cdk5 kinase activity, was increased in the dorsal root ganglia (DRG) and the spinal cord (SC) after peripheral-inflammation [12,14,15]. Inflammation induced by carrageenan injection [14] or by complete Freund's adjuvant (CFA) [15] in the hind paws of mice increased the mRNA and protein levels of Cdk5/p35 in nociceptive neurons with a subsequent increase in Cdk5 kinase activity. Furthermore, we also identified that the elevated Cdk5 activity phosphorylates transient receptor potential vanilloid 1 (TRPV1), a key receptor that modulates agonist-induced calcium influx in the neurons [16]. In addition, Cdk5-mediated phosphorylation of the δ-opioid receptor impaired receptor function and attenuated morphine anti-nociceptive tolerance [17]. Additionally, we found that inflammation triggers an increase in Cdk5 activity through activation of early growth response 1 (Egr-1) and p35 expression by TNF-α [13,18]. These findings suggest that Cdk5 plays an important role in the molecular mechanisms involved in pain signaling. To characterize a possible link between the analgesic effects of resveratrol and the role of Cdk5 in pain signaling, we set out to determine if resveratrol affects Cdk5 activity and, if it does, to characterize the mechanism by which it brings about this effect.

## Results

### Generation of p35 promoter-luciferase stable clones

**Figure 1:**
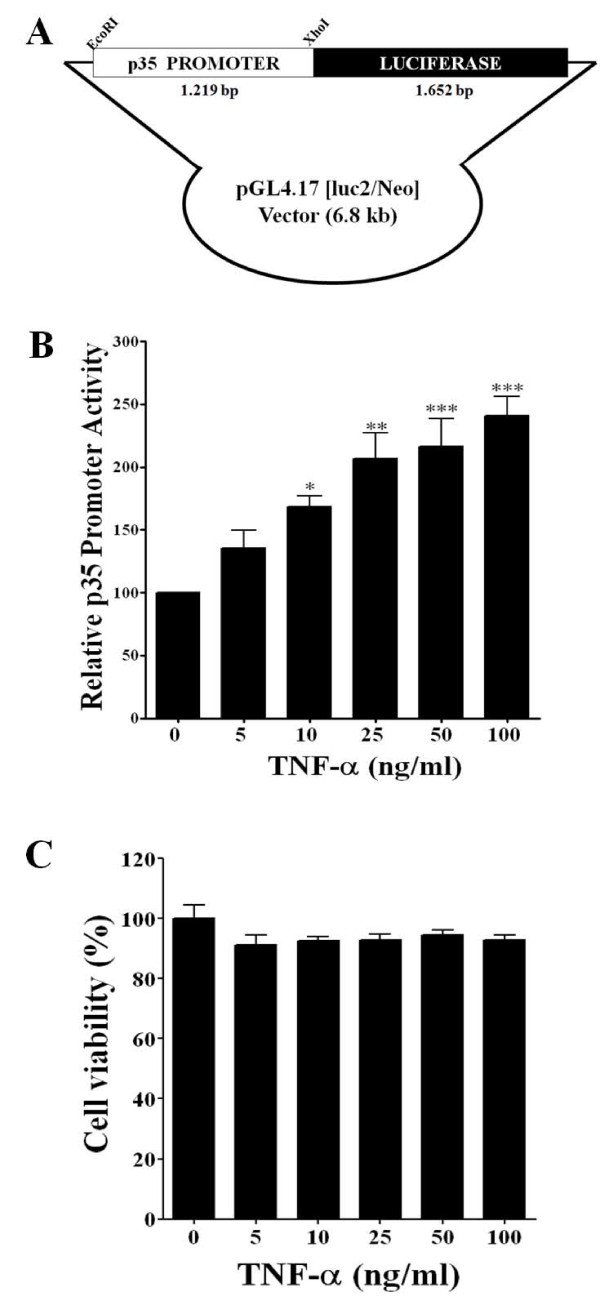
**Generation of p35 promoter-luciferase stable clones**. A) Schematic representation of the p35 promoter-luciferase vector (6.8 kb). It consists of a 1,219-bp fragment of a mouse p35 promoter cloned into a pGL4.17 (luc2/Neo) vector. B) TNF-α treatment at different concentrations increased p35 promoter activity in a dose-dependent manner at 24 h in stable clone C7 cells. C) At all levels of TNF-α concentrations that were tested, cell viability as measured by MTS assay was similar to the control. All data are presented as the mean and SEM (*n *= 3). * *p <*0.05, ** *p *< 0.01, ***p < 0.0001 (Dunett's test after ANOVA).

As reported earlier, we developed a cell-based assay using a transient transfection of PC12 cells with the p35 promoter-luciferase construct. With this assay we screened the effects of proinflammatory molecules on p35 promoter activity and found that TNF-α treatment of these cells significantly increased p35 promoter activity [18]. In order to establish a consistent cell-based assay, we generated several stable clones of the p35 promoter-luciferase in PC12 cells. Briefly, we cloned a 1,219-bp fragment of mouse p35 promoter [19] into the pGL4.17 (luc2/Neo) vector (Figure 1A). p35 promoter-luciferase vector was stably transfected into PC12 cells and subjected to G418 selection for four weeks, yielding 7 stable clones. As described in Materials and Methods, to test the functionality of these stable clones we analyzed the effects of TNF-α on their p35 promoter activity. All stable clones responded to TNF-α treatment (25 ng/ml), which resulted in increased p35-promoter activity, and based on this testing we selected the stable clone C7 for further experimentation (data not shown) and the effects of different concentrations of TNF-α on p35 promoter activity were further analyzed in the stable clone C7. TNF-α treatment increased p35 promoter activity in a dose-dependent manner, and at the highest concentration of 100 (ng/ml), p35 promoter activity increased by 250% compared to the control (Figure 1B). At all TNF-α concentrations tested, cell viability measured by MTS assay (as described in Materials and Methods) was similar to the control (Figure 1C).

### Resveratrol inhibits p35 promoter luciferase activity

**Figure 2:**
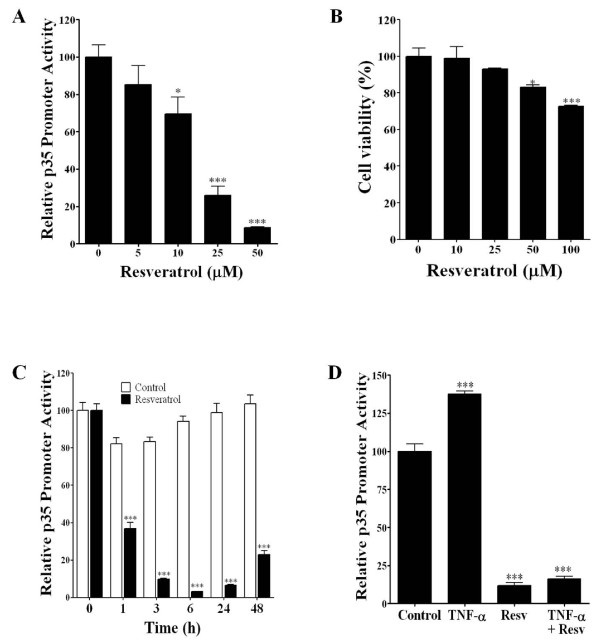
**Resveratrol inhibits p35 promoter activity**. A) Resveratrol treatment for 24 hr at different concentrations decreased p35 promoter activity in a dose-dependent manner in stable clone C7 cells. B) Cell viability was unchanged in up to 25 μM of resveratrol, but at 50 μM and 100 μM it was reduced significantly in comparison to the control. C) Resveratrol treatment (10 μM) decreased p35 promoter activity in a time-dependent manner. D) Stable clone C7 cells were treated with TNF-α (50 ng/ml), resveratrol (25 μM), and TNF-α (50 ng/ml) plus resveratrol (25 μM) during 24 h and p35 promoter activity was measured. Resveratrol blocked the TNF-α mediated increase in p35 expression. All data are presented as the mean and SEM (*n *= 4). *p <0.05, ***p < 0.0001 (Bonferroni test after ANOVA).

We analyzed the effects of different concentrations of resveratrol on p35 promoter activity in the stable clone C7, and we found that resveratrol significantly decreased p35 promoter activity in a dose-dependent manner (Figure 2A). Resveratrol treatment decreased p35 promoter activity at a concentration of 5 μM, which was followed by a linear decrease with higher concentrations of up to 50 μM. At a concentration of 25 μM, p35 promoter activity decreased by 70% compared to the control. We also evaluated the cell viability of stable clones by MTS assay and found that cell viability remained unchanged with up to 25 μM of resveratrol (Figure 2B). At 50 and 100 μM of resveratrol, cell viability was reduced by 16% and 27% compared to the control, respectively, which confirms earlier reports [20]. Next, we determined the time-course of decrease in p35 promoter activity with the resveratrol treatment. Stable clone C7 cells treated with resveratrol (10 μM) showed a time-dependent decrease in the p35 promoter activity (Figure 2C). Resveratrol treatment significantly decreased the p35 promoter activity, starting as early as 1 h after the treatment and reaching a further decrease at 6 h and 24 h. Furthermore, we tested whether resveratrol can block the increase in p35 promoter activity induced by TNF-α treatment. Stable clone C7 cells were treated with TNF-α (50 ng/ml) and resveratrol (25 μM) for 24 h, and this resulted in a significant decrease in p35 promoter activity (Figure 2D). Together, these results indicate that resveratrol treatment significantly decreases the p35 promoter activity in PC12 cells in a dose- and time-dependent manner, and also that resveratrol can block a TNF-α mediated increase in the p35 promoter activity.

### Resveratrol treatment significantly decreases p35 expression and Cdk5 activity in PC12 cells and DRG neuronal culture

**Figure 3:**
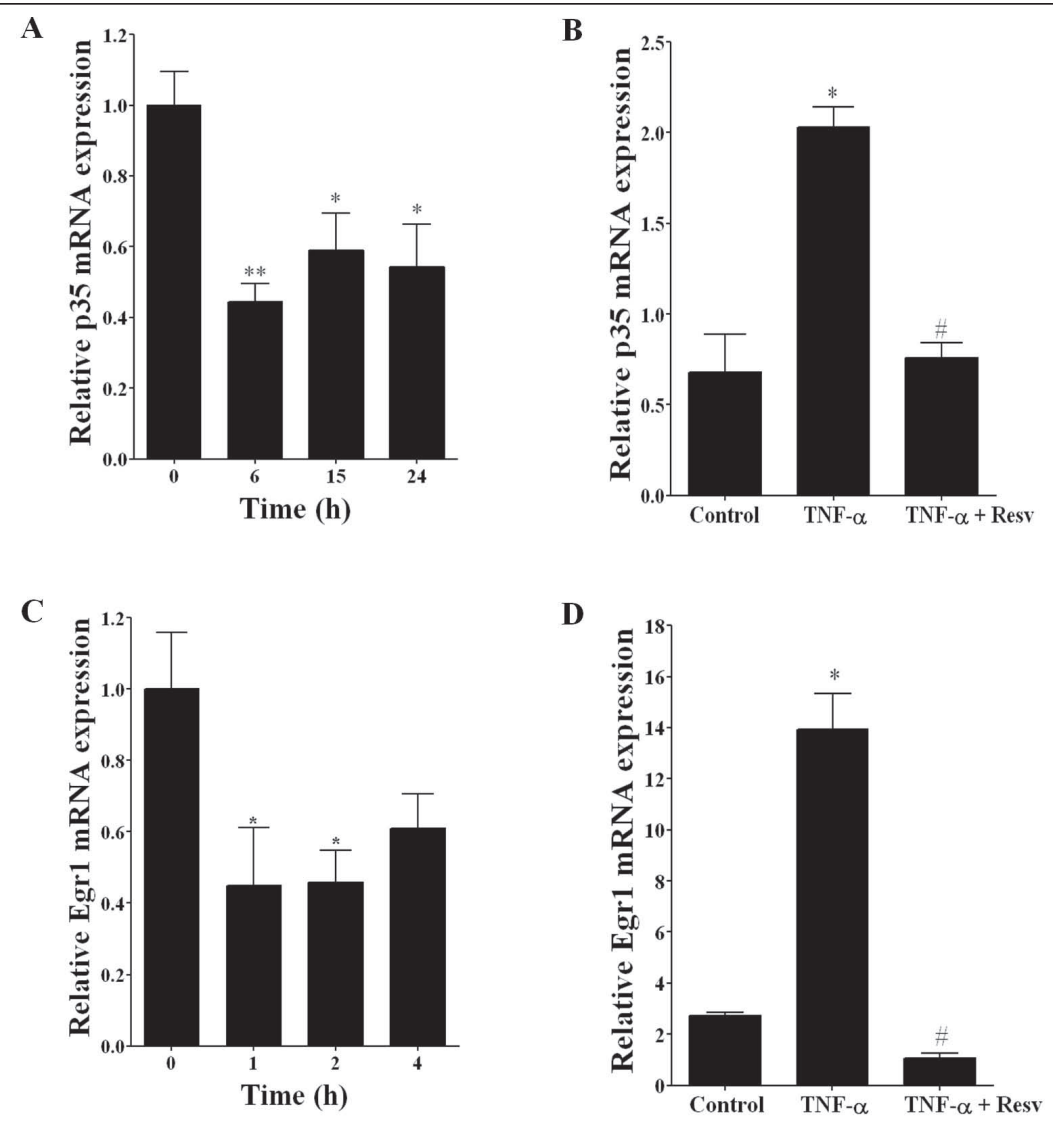
**Resveratrol treatment decreases p35 and Egr-1 mRNA levels and blocks the effects of TNF-α in PC12 cells**. A) Real-time RT-PCR analysis of p35 mRNA levels normalized against S29. Total RNA was obtained from PC12 cells treated with resveratrol (25 μM) for 0, 6, 15 and 24 h. After reverse transcription, we conducted real-time PCR with specific primers for p35 and S29. B) PC12 cells were treated with TNF-α (50 ng/ml), and TNF-α (50 ng/ml) plus resveratrol (25 μM) during a 3 h period. Resveratrol blocked the increase in p35 mRNA expression induced by TNF-α.C) Real-time RT-PCR analysis of Egr-1 mRNA levels normalized against S29. Total RNA was obtained from PC12 cells treated with resveratrol (25 μM) for 0, 1, 2 and 4 h. After reverse transcription, we carried out real-time PCR with specific primers for Egr-1 and S29. D) PC12 cells were treated with TNF-α (50 ng/ml), and TNF-α (50 ng/ml) plus resveratrol (25 μM) during a 1 h period. Resveratrol blocked the increase in Egr1 mRNA expression induced by TNF-α Bars are the mean and SEM of 3 independent experiments measured in triplicate. *p <0.05, **p<0.01 (Bonferroni test after ANOVA). #p <0.05 (Bonferroni test after ANOVA), # mean value for TNF-α treatment as compared to mean value for TNF-α plus resveratrol treatment.

**Figure 4:**
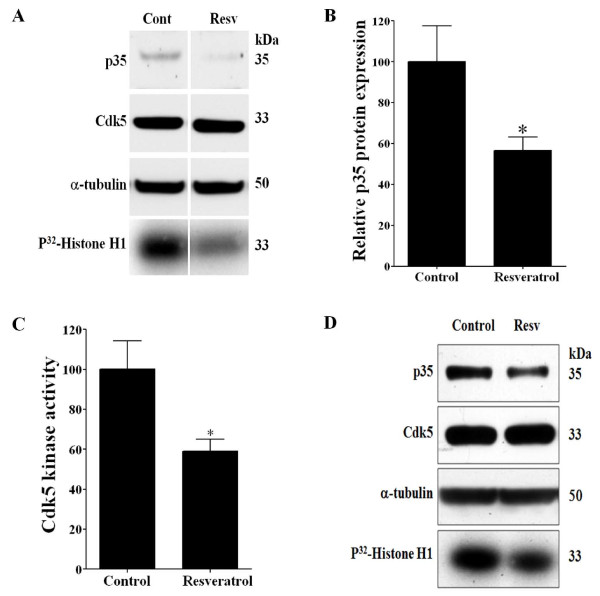
**Resveratrol treatment decreases p35 protein levels and Cdk5 kinase activity in PC12 cells and rat DRG neuronal cultures**. A) Representative Western blots analysis and Cdk5 kinase activity of protein from PC12 cells treated with resveratrol (Resv 25 μM) and untreated cells (Cont) during 24 h. Resveratrol treatment decreased p35 protein levels, and Cdk5 kinase activity. The levels of Cdk5 were unchanged and α-tubulin western blot was used as a loading control. B) Quantification of resveratrol treatment (25 μM) significantly decreased p35 protein expression in PC12 cells. C) Quantification of resveratrol treatment (25 μM) significantly decreased Cdk5 kinase activity in PC12 cells. D) Representative Western blots analysis and Cdk5 kinase activity of protein from rat DRG neuronal culture treated with resveratrol (Resv 25 μM) and untreated (Cont) during 24 h. Values are shown as mean and SEM of 3 independent experiments. * *p <*0.05 by using t-test.

To further examine the inhibitory effects of resveratrol on p35 promoter expression, we examined endogenous levels of p35 mRNA and protein at different time points following treatment with resveratrol. The level of p35 mRNA decreased significantly within 6 h after resveratrol treatment (25 μM) (Figure 3A), and the p35 protein level decreased significantly in PC12 cells (Figure 4A and 4B) and in rat DRG neuronal culture (Figure 4D) at 24 h following resveratrol treatment (25 μM). Moreover, we tested if resveratrol can block the increase in p35 mRNA induced by TNF-α. When PC12 cells were treated with TNF-α (50 ng/ml) and resveratrol (25 μM) for 3 h, this treatment blocked the TNF-α mediated increased in p35 mRNA (Figure 3B). On the other hand, Cdk5 mRNA and protein levels did not change significantly following resveratrol treatment (data not shown). Since the protein level of p35 is a limiting factor in Cdk5 kinase activity [21], we analyzed whether the resveratrol-mediated decrease in p35 expression results in decreased Cdk5 activity. We immunoprecipitated Cdk5 protein from the control and the resveratrol-treated cells and then assayed kinase activity by using histone H1 as a substrate [18]. After 24 h of resveratrol treatment (25 μM), Cdk5 kinase activity decreased significantly in PC12 cells (Figure 4A and 4C) and also in rat DRG neuronal culture (Figure 4D). We also found that Cdk5 activity was increased by TNF-α treatment (50 ng/ml), and that co-treatment with resveratrol (25 μM) blocked this increase (data not shown). In addition, we found that resveratrol is able to inhibit Cdk5 activity in mouse neuroblastoma N2a and rat neuroblastoma B104 cell lines (data not shown). Together, these results indicate that resveratrol treatment reduced expression of p35, which resulted in decreased Cdk5 kinase activity.

### Resveratrol treatment decreases Egr-1 mRNA and blocks TNF-α effects in PC12 cells

Because the p35 promoter region contains several putative sequence elements, including the binding site for transcription factor Egr-1 [19], we investigated whether resveratrol may regulate Egr-1 expression. Egr-1 mRNA levels were measured by real-time RT-PCR after resveratrol treatment, and we found that Egr-1 mRNA levels decreased after 1 h and 2 h of resveratrol treatment (25 μM) (Figure 3C). In addition, the Egr-1 mRNA levels increased after 1 h of TNF-α treatment (50 ng/ml), and resveratrol blocked this increase (Figure 3D).

### Resveratrol-mediated inhibition of p35 promoter activity through MAP kinases and NF-κB signaling pathways

**Figure 5:**
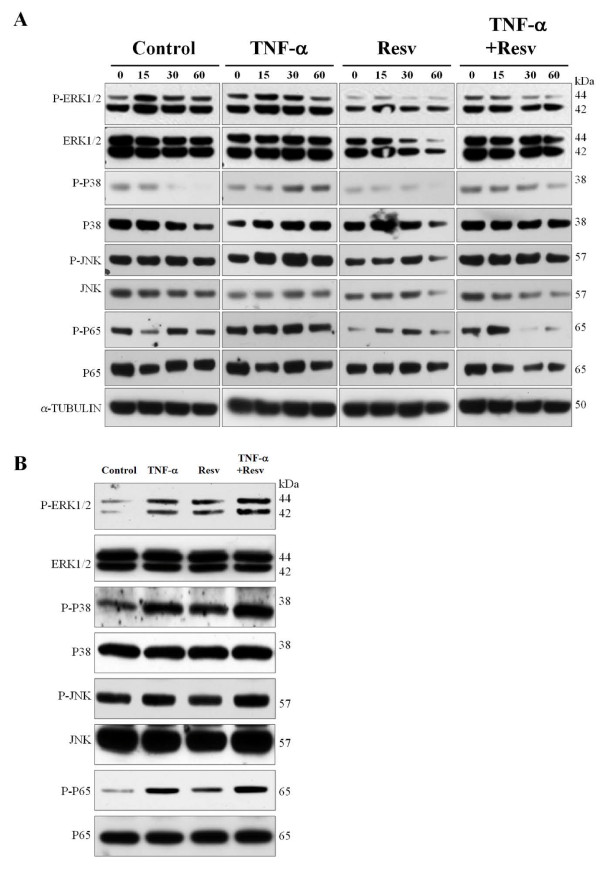
**Effects of MAP kinases and the NF-κB inhibitor on resveratrol-mediated inhibition of p35 promoter activity**. Western blot analysis of the activation of MAP kinases and NF-κB signaling pathways. Protein extract from PC12 cells untreated (Control), TNF-α treated (50 ng/ml), resveratrol treated (25 μM), and TNF-α (50 ng/ml) plus resveratrol (25 μM) treated for 0, 15, 30 and 60 min (A) and 24 h (B) were analyzed by Western blot using phospho-ERK1/2, total ERK1/2, phospho-p38 MAPK, total p38 MAPK, phospho-JNK, total JNK, phospho-p65 and total p65 antibodies.

Resveratrol is known to regulate several MAP kinase pathways, such as ERK1/2, p38 MAPK, JNK and NF-κB pathways [22-26]. We determined the regulation of MAP kinases and NF-κB pathways by resveratrol using Western blot analysis. We used phospho-antibodies to determine the activation of ERK1/2, p38 MAPK, JNK and NF-κB pathways at 0-60 min (Figure 5A) and at 24 h (Figure 5B) after resveratrol treatment (25 μM) of PC12 cells. ERK1/2 and NF-κB pathways were inhibited by resveratrol at 60 min; however, at 24 h after treatment, we observed higher levels of phospho-ERK1/2 and phospho-p65. Interestingly, p38 MAPK and JNK pathways remained unchanged after resveratrol treatment at each time point they were tested.

**Figure 6:**
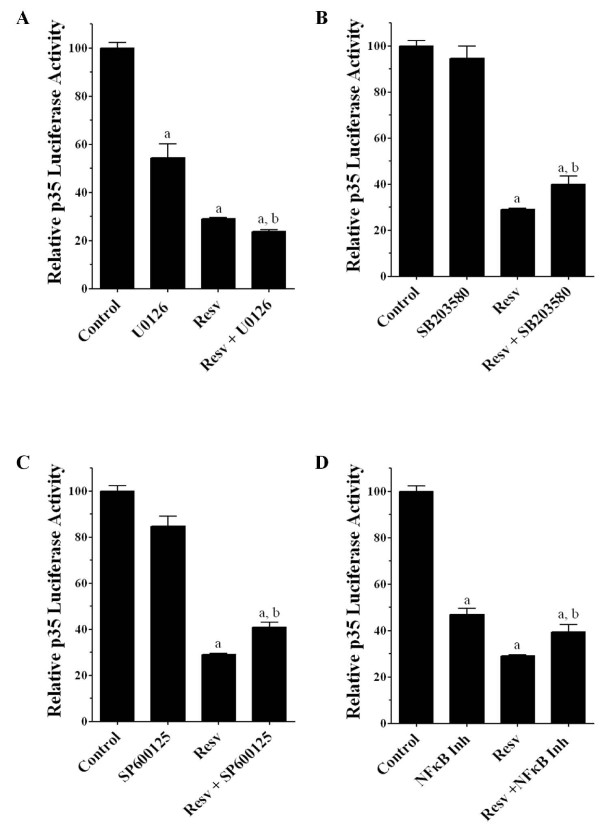
**Effects of MAP kinases and the NF-κB inhibitors on resveratrol-mediated inhibition of p35 promoter activity**. A) Stable clone C7 PC12 cells were untreated (control), treated with resveratrol (25 μM), MEK inhibitor U0126 (20 μM), and resveratrol (25 μM) plus U0126 (20 μM) during 24 h and luciferase activity was measured. Co-treatment of resveratrol plus U0126 had a further inhibitory effect over p35 promoter activity. On the other hand, B) p38 MAP kinase inhibitor (SB203580 20 μM), C) JNK inhibitor (SP600125 20 μM) and D) NF-κB inhibitor (1 μM), all of them significantly increased p35 promoter activity in cells treated with resveratrol (25 μM). All data are presented as the mean and SEM (*n *= 4). a, b *p <*0.05 (t-test). **a **mean control values compared with different treatments. **b **mean resveratrol values compared with resveratrol plus corresponding inhibitor.

We then examined the involvement of these pathways in resveratrol-mediated inhibition of p35 promoter activity, using specific inhibitors of MAP kinases and NF-κB with and without resveratrol, and measured p35 promoter activity in our stable clone C7 after 24 h of treatment. We tested a MEK inhibitor, U0126, which is an upstream regulator of ERK1/2 [27], as well as p38 MAPK inhibitor SB203580 [28], JNK inhibitor SP600125 [29], and an NF-κB inhibitor [30]. We found that U0126 (20 μM) alone or in combination with resveratrol (25 μM) significantly decreased p35 promoter activity (Figure 6A). This indicated that the ERK1/2 pathway is negatively regulated by resveratrol. In turn, p38 MAPK inhibitor SB203580 (20 μM), JNK inhibitor SP600125 (20 μM) and the NF-κB inhibitor (1 μM) significantly reversed the decrease in p35 promoter activity in cells treated with resveratrol (Figure 6B-D). This suggests that these pathways are positively regulated by resveratrol.

## Discussion

It is now known that Cdk5 plays an important role in pain signaling and is therefore considered a potential drug target for developing a new class of analgesics [12-16,18]. However, very few Cdk5-specific inhibitors have been identified so far and roscovitine remains the lead candidate, since it has somewhat better specificity although there were also some serious side effects reported in early clinical trials [31,32]. Most of these inhibitors bind to the ATP pockets of the kinases and therefore lack the specificity needed to inhibit Cdk5 alone [33]. In order to identify additional molecules that regulate Cdk5 with a better specificity, we developed a cell-based assay that measures p35 promoter activity and thereby p35 expression [18]. We previously reported that the p35 protein level is the limiting factor for Cdk5 activity in mouse brains [21], and this can be utilized to screen for molecules that would impact Cdk5 activity due to their effects on p35 expression. Using this cell-based assay, we successfully identified TNF-α as a major regulator of p35 expression and subsequently of Cdk5 kinase activity [18]. In our present work, we have improved this cell-based assay by establishing stable clones of PC12 cells transfected with p35-promoter-luciferase construct and tested these clones for their responses to TNF-α. We then selected one of these clones, C7, for further experiments. Our strategy is to use this assay to screen chemical libraries, in order to identify novel p35 inhibitors. However, before taking on the challenge of screening the chemical libraries, we wanted to use this cell-based assay to test the chemical that has been implicated as playing an analgesic role, and characterize its effects on Cdk5 activity. For that purpose, we selected resveratrol for testing and identified it as an effective inhibitor of p35 expression and Cdk5 activity. Resveratrol inhibited p35 expression, which resulted in a decrease in Cdk5 activity and blocked Cdk5 activation by TNF-α in PC12 cells and rat DRG neuronal culture. In addition, we investigated the mechanism underlying the inhibition of the p35 promoter by resveratrol. Here we demonstrate that, through inhibition of the ERK1/2 pathway and activation of the NF-κB pathway, resveratrol inhibits p35 expression in PC12 cells, thereby decreasing Cdk5 kinase activity. The inhibition of ERK1/2 by resveratrol leads to decreased Egr-1 expression, which then results in a reduction of p35 expression.

Resveratrol has been ascribed many therapeutic values, including analgesic properties [1-5]. Previous studies have demonstrated the analgesic effects of resveratrol in rodent models of inflammation and diabetic neuropathic pain [6,9,10,34-36]. Interestingly, anti-inflammatory effects of resveratrol are due to its ability to regulate levels of prostaglandins by inhibiting both COX-1 and COX-2 [8,11,37,38]. A single injection of resveratrol into the lateral ventricle of the brain reduced levels of COX-1 and COX-2 with a concomitant decrease in production of prostaglandins [8] and a decrease in the level of pain experienced by the rodents [34]. Resveratrol is also associated with activation of potassium channels [39], and is known to inhibit sodium currents in rat DRG neurons [40], although the molecular mechanism of this regulation is not entirely understood. Gupta and coworkers have recently demonstrated that the analgesic effects of resveratrol are mediated via opioidergic pathways, whereas the analgesic effects of morphine in the presence of resveratrol were significantly potentiated [41]. In conjunction with these findings, our studies have demonstrated a new molecular mechanism through which resveratrol can regulate nociception through inhibition of p35 expression and the subsequent decrease in Cdk5 activity. This is a significant finding in light of previous reports pointing to Cdk5 as an important key player in the regulation of pain signaling [12,13,18].

Numerous reports have indicated that resveratrol has different effects on MAP kinases and can inhibit the activation of NF-κB signaling in certain cells or in a tissue-specific manner [22-26]. Resveratrol can either inhibit or activate ERK1/2 signaling pathways depending on the cell types or the doses used [23,24,42,43]. For example, resveratrol at lower doses (up to 10 μM) is known to activate the ERK1/2 pathway and, whereas, at higher doses (50-100 μM) it can inhibit the ERK1/2 pathway in SH-SY5Y human neuroblastoma cells [24]. Our study shows that resveratrol (25 μM) reduced the phospho-ERK1/2 level at 1 h, whereas it increased this level at 24 h. In addition, treatment with the MEK inhibitor (U0126 [27]) and resveratrol produced a significant decrease in p35 promoter activity. Moreover, resveratrol treatment blocked TNF-α mediated activation of p35 expression and the subsequent increase in Cdk5 activity, possibly because of decreased p35 expression due to the down-regulation of the ERK1/2 pathway by resveratrol. On the other hand, resveratrol also suppresses phosphorylation and subsequent degradation of IκB, thereby inhibiting activation of the NF-κB signaling pathway. Similarly, it also suppresses TNF-α-induced activation of NF-κB as well [37,42,44,45]. Our results are in partial agreement with these reports, since we found that resveratrol decreased phospho-p65 at 1 h, although it did not block the TNF-α-mediated increase of phospho-p65 in PC12 cells. Surprisingly, resveratrol treatment increased the phospho-p65 level at 24 h, which is in agreement with the reported increase of p35 promoter activity following treatment with resveratrol and the NF-κB inhibitor, compared with resveratrol treatment alone. These results suggest that resveratrol positively regulates the NF-κB pathway in PC12 cells, which in turn decreases p35 expression [18]. Resveratrol regulates p38 MAPK and JNK pathways differently in different systems [25,42,46,47]. Interestingly, our study shows that resveratrol has no effect on p38 MAPK or JNK phosphorylation, as determined by Western blot analysis. However, we also observed that treatment with the p38 MAPK inhibitor (SB203580 [28]) or the JNK inhibitor (SP600125 [29]), in the presence of resveratrol, increased p35 promoter activity as compared with resveratrol treatment alone. These results suggest a positive regulation of the p38 MAPK and JNK pathways by resveratrol, which in turn could decrease p35 expression.

Resveratrol treatment can increase Egr-1 expression at different time points through the ERK1/2-dependent mechanism [48-50]. In contrast, our study shows that resveratrol treatment decreased Egr-1 mRNA expression at an early time point, and also blocked the TNF-α-mediated increase of Egr-1 in PC12 cells. This discrepancy might be due to variations in the concentrations and experimental conditions used for different studies.

## Conclusions

**Figure 7:**
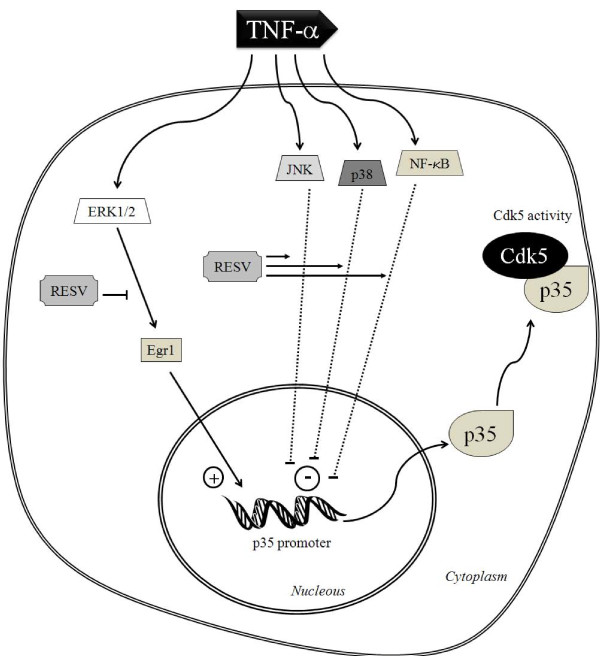
**Proposed resveratrol-mediated regulation of p35 expression and Cdk5 activity**. Resveratrol (RESV) reduces Cdk5 activity through inhibition of p35 expression. The inhibitory effects of resveratrol on p35 expression could be brought about at several levels, inhibition of ERK1/2 signaling pathway could affect Egr-1 expression, or activation of the p38 MAPK, JNK and NF-κB signaling pathways could downregulate p35 promoter activity.

In summary, our study demonstrates that resveratrol regulates key components of signal transduction pathways that affect p35 promoter activity. Most importantly, resveratrol blocks the TNF-α-mediated increase in p35 promoter activity, thereby reducing p35 expression and subsequent Cdk5 kinase activity (Figure 7). This new molecular mechanism adds to the known analgesic effects of resveratrol brought about mainly by its regulation of COX-1 and COX-2. Finally, these findings validate our cell-based assay for its use in the high throughput screening (HTS) of chemical libraries, used to identify potential analgesics based on their ability to reduce Cdk5/p35 activity for the effective treatment of pain.

## Materials and methods

### Materials

Resveratrol, mouse recombinant TNF-α, histone H1, SP600125 and α-tubulin antibody were obtained from Sigma (St. Louis, MO). SB203580, NGF and NF-κB inhibitor were obtained from Calbiochem (San Diego, CA). Protein quantification reagents were obtained from Bio-Rad Laboratories (Hercules, CA), and enhanced chemiluminescence reagents for Western blot analysis were purchased from Thermo Scientific (Rockford, IL). Luciferase Reporter Assay System and CellTiter 96 AQueous One solution Cell Proliferation Assay (MTS) were obtained from Promega (Madison, WI).

### Antibodies

Antibodies to Cdk5, p35, JNK, phospho-JNK, and secondary antibodies (HRP-conjugated goat anti-mouse, anti-rabbit antibodies) were obtained from Santa Cruz Biotechnology, Inc. (Santa Cruz, CA). Antibodies to ERK1/2, phospho-ERK1/2, p38 MAPK, phospho-p38 MAPK, NF-κB p65, phospho-NF-κB p65 and U0126 were obtained from Cell Signaling Technology (Beverly, MA).

### Cell culture

PC12 cells (derived from pheochromocytoma of rat adrenal medulla) were obtained from American Type Culture Collection (Rockville, MD). PC12 cells were cultured in Dulbecco's modified Eagle's medium (DMEM, Invitrogen, Carlsbad, CA), supplemented with 10% fetal bovine serum (Hyclone Laboratories, Logan, UT), penicillin and streptomycin (Invitrogen, Carlsbad, CA).

### Embryonic rat DRG neuronal culture

DRGs were harvested from 15-day embryonic Sprague-Dawley rats. The tissue was dissociated in Ca^+2^/Mg^+2^-free Hank's balanced salt solution (HBSS, Invitrogen, Carlsbad, CA) containing 0.5 U/ml Liberase Blendzyme 3 (Roche Applied Science) for 1 h at 37°C. The DRGs were then triturated in complete growth medium [MEM, 5% heat-inactivated horse serum (Invitrogen, Carlsbad, CA), 50 ng/ml 2.5S murine NGF, N3 supplement] with 50 μg/ml DNase using a fire-polished glass pipette. The suspension was enriched for neurons by spinning on a two-layer, 30%:50% Percoll gradient (GE Healthcare Biosciences) at 800 × g for 20 min. The Percoll was removed by diluting with HBSS then spinning down the cells at 400 × g for 5 min. Cells were re-suspended in complete growth medium then plated onto poly-D-lysine/laminin coated plates. DRGs were cultured 4-6 days before measurement, refreshing the medium every 2 days. On the first day after plating, 10 μM fluorodeoxyuridine was added to halt mitosis of dividing cells in combination with 20 μM uridine to preserve RNA synthesis. Primary DRG cultures at this stage were treated with resveratrol (25 μM) or vehicle for 24 h. Proteins were extracted and analyzed by Western blotting.

### Preparation of the p35 promoter-luciferase reporter plasmid

We constructed a p35 promoter-luciferase vector by inserting a 1,219-bp mouse p35 promoter into the pGL4.17 (Luc2/Neo) vector from Promega (Madison, WI). Briefly, pBluescript II SK(-) p35 promoter vector [19] was digested with *Xba*I and *Xho*I, and a 1,219-bp fragment containing the p35 promoter was cloned between the *Nhe*I and *Xho*I sites of the pGL4.17 vector.

### Stable transfection and reporter activity assays

p35 promoter-luciferase vector was stably transfected into PC12 cells using Lipofectamine™ LTX and Plus™ Reagent (Invitrogen, Carlsbad, CA). Transfected cells were subjected to drug selection by culturing them with geneticin^® ^(500 μg/ml) (G418, Invitrogen, Carlsbad, CA) for four weeks, and then several stable clones were established. The p35 promoter-driven luciferase activity was determined using the Luciferase® reporter Assay system from Promega. As reported earlier [18], we tested several concentrations of TNF-α (0, 5, 10, 25, 50, and 100 ng/ml) during a 24 h period to determine which stable clone responded better to TNF-α. Based on this testing, we selected the stable clone C7 for further experiments. We tested luciferase activity in stable clone C7 at different concentrations of resveratrol (0, 5, 10, 25, and 50 μM) and different time points (0, 1, 3, 6, 24, and 48 h). In the second set of experiments, stable clone C7 cells were treated with inhibitors of MAP kinases (U0126, SB203580, and SP600125 from Sigma) or NF-κB inhibitor prior to resveratrol treatment for 24 h.

### MTS Assay for Cell viability

Stable p35-promoter PC12 cells plated in 96-well plates were serum-starved for 1 h, and treated with TNF-α (0-100 ng/ml), resveratrol (0-100 μM), MAP kinases inhibitors (U0126, 20 μM; SB203580, 20 μM; and SP600125, 20 μM) or an NF-κB inhibitor (1 μM) in serum-free DMEM for 24 h. Cells were then incubated with CellTiter 96 AQueous (MTS) solution (20 μl) from Promega (Madison, WI). After 1 h of incubation with CellTiter 96 AQueous solution, colored MTS products in the supernatant were transferred into 96-well microtiter plates and absorbance at 490 nm was determined on MicroPlate Reader Safire (Tecan, Switzerland).

### RNA isolation and real-time RT-PCR

PC12 cells were grown in 6-well plates and were incubated with a vehicle, TNF-α (25 ng/ml), resveratrol (10 μM) and TNF-α (50 ng/ml) plus resveratrol (25 μM) for several time points (0, 1, 2, 3, 4, 6, 15 and 24 h) in serum-free medium. After discarding the growth medium, total RNA was isolated from the cells using TRIzol^® ^reagent (Invitrogen, Carlsbad, CA) according to the manufacturer's instructions. Following TURBO DNA-free (Ambion, Austin, TX) digestion of the total RNA sample, oligo (dT) primed synthesis of cDNA from 3 μg of total RNA was made using SuperScript™III Reverse Transcriptase (Invitrogen, Carlsbad, CA) to remove contaminated genomic DNA. For detection of Egr-1 and p35 mRNA, we used real-time PCR, and the following reaction mixture was used for these PCR samples: 1 × IQ™ Sybr^®^Green Super Mix (Bio-Rad, Hercules, CA), 100-200 nM of each primer and 1 μl of cDNA. cDNA was amplified and analyzed in triplicate using Opticon Monitor Chromo 4 (Bio-Rad, Hercules, CA). The following primers were used to amplify and measure the amount of mouse mRNA by real-time RT-PCR: Egr-1 S: 5'-CCC TTC CAG GGT CTG GAG AAC CGT-3', Egr-1 AS: 5'-GGG GTA CTT GCG CAT GCG GCT GGG-3', p35 S: 5'-GCC CTT CCT GGT AGA GAG CTG-3', p35 AS: 5'-GTG TGA AAT AGT GTG GGT CGG C-3'. The gene expression level was normalized to *S29*. S29 S: 5'-GGA GTC ACC CAC GGA AGT TCG G-3' and S29 AS: 5'-GGA AGC ACT GGC GGC ACA TG-3'. Real-time PCR reactions were run in triplicate and repeated three times.

### Immunoblot Analysis

PC12 cells and rat DRG neuronal cells were lysed in T-PER buffer (Pierce, Rockford, IL) with protease inhibitor cocktail tablets and phosphatase inhibitor cocktail tablets, PhosSTOP (Roche Diagnostic, Indianapolis, IN). Protein concentration of the supernatant was determined using Bradford Protein Assay (Bio-Rad, Hercules, CA). Proteins were separated by 4-12% SDS-PAGE gels and transferred to nitrocellulose membranes (Invitrogen, Carlsbad, CA). The membranes were soaked in a blocking buffer (5% nonfat dry milk in phosphate-buffered saline with 0.05% Tween-20 (PBST)) for 1 h at room temperature, and then incubated overnight at 4°C with the appropriate primary antibody diluted in the blocking buffer. The membranes were washed in PBST and incubated for 1 h at room temperature with the secondary antibodies diluted in blocking buffer. Immunoreactivity was detected by SuperSignal West Pico or Dura Chemiluminescent Substrate (Thermo Scientific, Rockford, IL). Membranes were stripped for 15 min at room temperature with Re-blot Plus Strong Solution (Chemicon, Temecula, CA) and retested with α-tubulin antibodies to normalize for protein loading. The optical densities of the bands were quantified using an image analysis system with *Scion Image Alpha *4.0.3.2 software (Scion Corporation, Frederick, MD).

### Cdk5 Kinase Activity Assay

Cdk5 kinase activity was measured as described [14]. Briefly, 150-250 μg of protein from PC12 cells and rat DRG neuronal culture treated with vehicle or resveratrol (25 μM) were dissolved in T-PER buffer and immunoprecipitated with 4 μg of anti-Cdk5 antibody C8 (Santa Cruz, CA). Immunoprecipitated proteins (IP) were washed 3 times in cold PBS, and 2 times in kinase buffer [20 mM Tris HCl (pH 7.4), 10 mM MgCl_2_, 1 mM EDTA, 10 μM NaF and 1 μM Na_2_VO_3_]. Then IP were mixed with the kinase assay mixture [100 mM Tris HCl (pH 7.4), 50 mM MgCl_2_, 5 mM EDTA, 50 μM NaF, 5 μM Na_2_VO_3 _and 5 mM DTT], using Histone H1 (1 μg/μl) as a substrate, and the kinase activity was quantified as described [14].

### Statistical Analysis

All experiments were performed a minimum of 3 times. Statistical evaluation was done with *GraphPad Prism *software, version 4.0 (GraphPad, San Diego, CA). Significant differences between the experiments were assessed by univariate ANOVA (more than 2 groups) or unpaired t-tests (2 groups). ANOVA was followed by t-tests using a Bonferroni α-correction or Dunett's test, where α was set to 0.05.

## Abbreviations

Cdk5: cyclin-dependent kinase 5; TNF-α: tumor necrosis factor-alpha; ERK1/2: extracellular-signal regulated kinase1/2; Egr-1: early gene response 1; TRPV1: transient receptor potential vanilloid 1; DRG: dorsal root ganglia; CFA: complete Freund's adjuvant.

## Competing interests

The authors declare that they have no competing interests.

## Authors' contributions

EU designed, carried out the experiment and data analysis, and contributed to the manuscript writing. AT and JK assisted in conducting experiments, MI contributed to manuscript preparation and AK designed, contributed to manuscript writing and supervised work. All authors read and approved the final manuscript.
